# Biogenic chlorapatite nanoparticles modulate stress-responsive genes and antioxidant defense to alleviate drought stress in Cowpea

**DOI:** 10.1186/s12870-026-08589-5

**Published:** 2026-04-11

**Authors:** Doaa E. Elsherif, Mai A. El-Esawy, Aya E. Khalifa

**Affiliations:** 1 Botany Department, Faculty of Science, Tanta University, Tanta, 31527 Egypt; 2 Zoology Department, Faculty of Science, Tanta University, Tanta, 31527 Egypt

**Keywords:** Drought, Nanoparticles, Chlorapatite, Antioxidants, Gene expression, Cowpea (*Vigna unguiculata*)

## Abstract

**Background:**

Drought stress is a significant environmental challenge that adversely affects plant health and productivity. Nano-fertilizers, particularly green synthesized nanoparticles, offer a promising and eco-friendly strategy to enhance plant resilience and productivity under stress.

**Results:**

Biogenic chlorapatite nanoparticles (CAp NPs) were synthesized using pectin extracted from *Ficus elastica* leaves racterized by UV–Vis spectroscopy, TEM, and FT-IR, confirming their spherical morphology, average diameter of 18.5 ± 3.7 nm. Cowpea (*Vigna unguiculata*) was irrigated with CAp NPs at various dosages (0, 50, 100, and 200 mg/L) to mitigate the harmful effects of drought stress (30% water field capacity) on 15-day-old cowpea plants. Under drought stress, cowpea plants showed significant reductions in growth parameters and increases in oxidative stress markers, whereas CAp NP application (50–200 mg/L) notably enhanced growth, reduced oxidative damage, and improved osmoprotectant (soluble sugars, proteins), nonenzymatic (phenolics, flavonoids, ascorbic acid, reduced glutathione) and antioxidant enzyme activities. The results also revealed that CAp NP treatments significantly enhanced soil microarthropod and key soil properties, including pH, moisture, EC, organic matter, available phosphorus, and total nitrogen. Additionally, CAp NPs modulated gene expression of key stress-responsive genes (*catalase2 (CAT2), superoxide dismutase1 (SOD1), dehydration-responsive element binding protein (DREB),* and *magnesium chelatase (CHLH*) and influenced oribatid mite diversity, with the highest diversity and abundance observed at the highest CAp NP dose.

**Conclusion:**

These findings highlight the potential of CAp NPs as a promising nano-enabled strategy for sustainable agriculture in water-limited environments. They likely function as a multifaceted stress protectants by providing essential nutrients, boosting antioxidant systems, and improving water-use efficiency.

## Introduction

Climate change acts as a powerful amplifier of drought stress, severely impacting plant health and survival. Rising global temperatures increase atmospheric evaporation, drying out soils more rapidly and forcing plants to close their stomata to conserve water [1]. This survival tactic comes at a high cost: it shuts down photosynthesis and halts growth, leading to weakened systems, reduced agricultural yields, and widespread forest decline [2, 3]. The FAO reported that drought caused over 34% of crop and livestock production losses, making it the most destructive climate-related threat to agriculture [4]. Moreover, water deficit triggers stomatal closure to minimize transpirational water loss, which simultaneously restricts carbon dioxide uptake and leads to a decline in photosynthetic activity, growth inhibition, and wilting [5]. Drought induces oxidative stress through the accumulation of reactive oxygen species (ROS), causing damage to cellular structures such as membranes, proteins, and nucleic acids [6, 7]. In response, plants activate defense mechanisms, synthesizing osmoprotectants like proline and glycine betaine to maintain cellular turgor, increasing antioxidant enzyme to scavenge ROS, and altering hormone signaling, particularly abscisic acid, to regulate stress adaptation [8, 9]. Plants activate complex stress-response pathways, upregulating genes that encode for protective proteins like dehydrins and osmoprotectants (e.g., proline), as well as transcription factors that regulate antioxidant defense systems [10, 11]. These molecular changes represent an adaptive effort to maintain cellular homeostasis, though prolonged drought exceeds tolerance limits and may ultimately compromise viability and yield [12].

The applications of nanotechnology in agriculture include the use of nanofertilizers and nanopesticides, which can monitor nutrients, stimulate plant growth and productivity, and increase resilience to stress [13, 14]. The distinctive physicochemical properties of nanofertilizers, such as their enhanced surface area and reactivity, enable the targeted application of nutrients and bioactive compounds [15]. Nanofertilizers also lessen oxidative stress while stimulating biochemical activity, such as elevating proline and chlorophyll levels [7]. Additionally, these nanofertilizers help plants maintain water balance, regulate toxic salt levels, and decrease the accumulation of damaging agents, including malondialdehyde and hydrogen peroxide [16]. Furthermore, nanofertilizers are vital for maintaining ionic equilibrium in plants under stress [14, 17]. As a result, employing nanofertilizers to improve crop resilience under stress has gained significant prominence in contemporary research.

Green nanoparticles synthesized via plant extracts offer a sustainable approach to mitigating plant stress [18]. A notable example is Chlorapatite (CAp), with the chemical formula Ca_5_ (PO_4_)_3_Cl. This biocompatible compound possesses a high adsorption capacity, making it an ideal candidate for environmental remediation and agricultural applications [19]. It can be prepared through green routes using natural sources like eggshells or plants [20]. Green-synthesized apatite nanoparticles are important in agriculture for enhancing nutrient delivery, soil water retention, and plant stress tolerance [21]. Their eco-friendly synthesis and nano-scale structure improve phosphorus and calcium availability, support root growth, and reduce oxidative damage under plant stress [22]. Their nanosize enables enhanced nutrient uptake and bioavailability, reducing the need for conventional fertilizers. This offers a sustainable approach to boost crop resilience and productivity [23]. Research indicates that apatite nanoparticles, especially those synthesized through green methods, are relatively safe for microorganisms and soil fauna at certain concentrations [24]. They may even contribute to improved plant growth and soil fertility when used at controlled concentrations.

Cowpea (*Vigna unguiculata*), commonly known as black-eyed pea or lobia, is an important legume crop cultivated widely in tropical and subtropical regions due to its high nutritional value and adaptability to harsh environments [25]. It is a rich source of plant-based proteins, vitamins, and minerals, making it a staple food in many developing countries [26, 27]. Agronomically, cowpea plays a vital role in sustainable farming systems because of its ability to fix atmospheric nitrogen, thereby improving soil fertility and reducing the need for chemical fertilizers [28]. Additionally, cowpea contributes to food security by providing grains for human consumption and foliage for animal feed, making it a dual-purpose crop with significant socio-economic importance [27]. To bridge this knowledge gap, this study investigated the efficacy of biogenic chlorapatite nanoparticles (CAp NPs) as nanofertilizers in alleviating drought stress in cowpea and to elucidate the underlying physiological, biochemical, and molecular responses.

## Materials and methods

### Materials

The chemical materials used in this study include, Calcium chloride dehydrate (CaCl₂0.2H₂O), diammonium hydrogen phosphate ((NH₄)₂HPO₄), ethanol (95%) were purchased from Sigma Aldrich. All chemicals were of high purity, analytical grade. Distilled water were used for the biogenic synthesis of chlorapatite nanoparticles (CAp NPs).

### Green synthesis of chlorapatite nanoparticles (CAp NPs)

*Ficus elastica* was collected from the garden of Faculty of Science, Tanta University, Egypt (30° 48′ 1″ N, 30° 59′ 21″ E). The plants were formally identified by Dr. Esraa E. Ammar, lecturer at Tanta University Herbarium (TANE), Egypt. Fresh leaves were thoroughly washed with distilled water. For extract preparation, 1 g of ground leaf material was boiled in 25 mL of distilled water at 60 °C, then allowed to cool. The mixture was subsequently centrifuged at 4000 rpm for 15 min. The obtained precipitate was suspended in double distilled water (solid– liquid ratio of 1:25, w/v), then the suspension was stirred at 60 °C for 4 h. The resulting slurry was cooled to room temperature and filtered through Whatman paper No.1. The residue was resuspended in double-distilled water at the same ratio (1:25 w/v) and the extraction process was repeated. The supernatants from both extractions were combined, and water-soluble pectin was precipitated by adding four volumes of ethanol. The final precipitate was collected by filtration, dried at 40 °C, and stored for subsequent use.

**Fig. 1:**
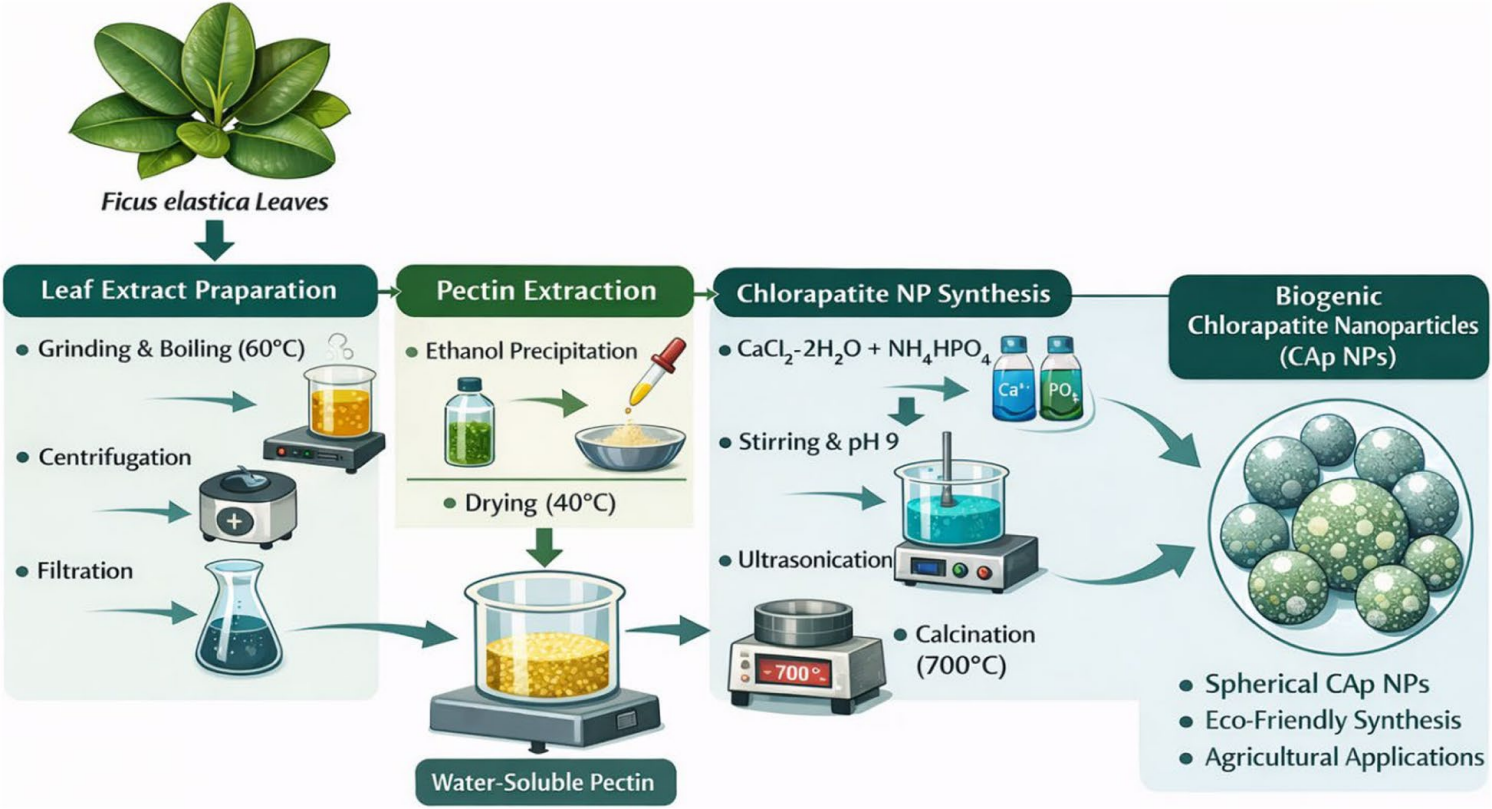
Schematic illustration of the green synthesis of chlorapatite nanoparticles (CAp NPs) mediated by pectin extracted from *Ficus elastica* leaves

Chlorapatite nanoparticles (CAp NPs) were synthesized via the pectin-mediated precipitation route described in [29], with specific modifications to stabilize the chloride-containing phase. Briefly, 0.01 wt% pectin was dissolved in 50 mL of distilled water and heated to 60 °C. To this solution, 0.05 M CaCl₂·2H₂O was added and stirred for 1 h to facilitate cooperative interaction. Subsequently, a 0.03 M (NH₄)₂HPO₄ solution was added dropwise to the mixture under vigorous magnetic stirring. This reaction was continued for 3 h, resulting in a pale yellow-green suspension at pH 9 and stirred for 24 h. The resulting precipitate was collected, dried in a hot-air oven at 80 °C, and then calcined. The dried powder was finally sintered at 700 °C for 6 h to obtain crystalline CAp NPs (Fig. 1).

### Characterization of green synthesized CAp NPs

Initial confirmation of synthesis was obtained using a UV–visible spectrophotometer (Shimadzu 240). The crystalline phase was identified by X-ray diffraction (XRD) analysis (GNR-APD 2000 Pro). The morphology and particle size were analyzed by high-resolution transmission electron microscopy (HR-TEM, JEOL JEM-2100, operated at 200 kV). For TEM imaging, the nanoparticle suspension was first sonicated for 20 min (Crest Ultrasonics Corp.). A droplet of the suspension was then placed on a carbon-coated copper grid, allowed to dry, and subsequently imaged. Finally, the chemical composition and functional groups present in the CAp NPs, as well as in the *F. elastica* leaf extract used for synthesis, were identified using a Fourier-transform infrared (FT-IR) spectrophotometer (Bruker Tenor 27).

### Plant materials and experimental design

The soil used for the experiment was collected from a garden at Faculty of Science, Tanta University (30°48′04.5′′N 30°59′35′′E), Al-Gharbiya Governorate, Egypt. After collection, soil microarthropods were extracted from soil using modified Berlese-Tullgren funnels.

Cowpea (*Vigna unguiculata* cv. Kareem 7) seeds were procured from the Gemmeiza Agriculture Research Station, El-Gharbia, Egypt. Prior to planting, the seeds were surface-sterilized by immersion in 1% NaOCl for 15 min, followed by thorough rinsing with sterile distilled water. Five sterilized seeds were sown in each cultivation pot (15 cm diameter × 8 cm depth) containing 1 kg of pre-mixed, air-dried soil (sand:clay ratio of 1:2, v/v). The experiment was conducted in June at the experimental garden of the Faculty of Science, Tanta University, Egypt. The environmental conditions were characterized by high temperatures typical of Egyptian summer, with an average daily temperature ranging from 33–36 °C, an average relative humidity of 50–60%, and a maximum photoperiod exceeding 13–14 h of light. During the initial 15-day establishment period, the control group was irrigated regularly with tap water. The other pots were irrigated with 30% of water field capacity as negative control (drought stress) from day one of cultivation. To determine the gravimetric water content (WC) at field capacity (FC), soil samples were weighed both before and after being dried at 105 °C for a 24-h period. Using this baseline, drought conditions were established and regulated at 30% FC. To ensure precision, soil moisture levels were not merely estimated but were actively tracked and regulated using a Capacitive Soil Moisture Sensor Module (v1.2, corrosion-resistant probe, DC 3.3–12 V, analog output, compatible with Arduino). After 3 days of cultivation, the plants were subjected to irrigation with biogenic CAp NPs twice weekly in four groups as follows: drought stress + 0 mg/L CAp NPs, drought stress + 50 mg/L CAp NPs, drought stress + 100 mg/L CAp NPs and drought stress + 200 mg/L CAp NPs each treatment represented by three pots. Fifteen days later, the morpho-physiological features were measured. Additionally, soil micro-arthropods were extracted from each set using modified Berlese-Tullgren funnels. Oribatid mites were isolated from other soil microarthropods under a microscope, preserved, and subsequently identified to species [30].

### Growth parameters

To assess growth performance, harvested plants were thoroughly rinsed. Morphological measurements, shoot length (cm) and root length (cm), were subsequently taken, and biomass was determined by recording the fresh (g) and oven-dry weights (g) of the entire plant.

### Soil chemical analysis

Sampling was performed for each treatment at initiation (day 0) and after 15 days. The samples were placed on a polythene sheet for 48 h to dry in air, passed through a 2.0 mm sieve, mixed uniformly and used for future analysis. Following [31], a 1:5 soil/water extract was used to determine pH, moisture percentage, and electrical conductivity (EC). Soil organic matter (OM) content was determined by the loss-on-ignition method. Samples were ignited in a muffle furnace at 550 °C for 4 h, and the loss in mass was calculated as a percentage of the original dry soil weight [32]. Organic carbon (OC) content was estimated as 50% of the OM value, based on a standard conversion factor [33]. Total nitrogen was analyzed according to [34]. Soil samples were first digested in a mixture of nitric acid and hydrogen peroxide prior to distillation and titration. Available phosphorus was extracted with a NaHCO₃ solution and quantified by colorimetric analysis, following the procedure described by [35].

### Oxidative stress biomarkers

Fresh leaf samples were analyzed for hydrogen peroxide (H₂O₂) and malondialdehyde (MDA) content to assess oxidative stress. For H₂O₂, tissue was extracted with a trichloroacetic acid (TCA)/KI/phosphate buffer solution and measured spectrophotometrically at 390 nm [36]. For MDA, a marker of lipid peroxidation, thiobarbituric acid (TBA) assay was performed [37]; absorbance readings at 532 nm and 600 nm were used to calculate the concentration in n mol/g fresh weight.

### Osmolyte contents

Total soluble sugars were assessed via the phenol–sulfuric acid method [38], with spectrophotometric measurements at 490 nm (UVISCO model V-1200). Results are expressed as mg/g dry weight.

The total soluble protein content was determined according to the Bradford method [39]. Following the assay, the absorbance was recorded at 595 nm. The protein concentration was calculated in mg/g dry weight.

### Estimation of non-enzymatic antioxidants

As reported by [40], fresh leaves were dried to estimate total phenolic content. The absorbance was assessed at 650 nm with a gallic acid standard curve (0–1 mg l⁻^1^, R2 = 0.9942). Total flavonoids were assayed via an AlCl₃-based method [41]and quantified against a quercetin standard (0–1 mg l⁻^1^, R2 = 0.9848) at 417 nm. Ascorbic acid was extracted with 5% sulfosalicylic acid and measured at 660 nm [42]. Reduced glutathione was determined according to [43]by measuring absorbance at 412 nm.

### Extraction and assay of enzymatic antioxidants

Enzyme extracts were prepared by homogenizing 0.5 g of fresh leaf tissue in 8 mL of cold 0.1 M phosphate buffer (pH 7.0) for subsequent enzyme activity assays. Polyphenol oxidase (PPO) activity was quantified by monitoring purpurogallin formation at 420 nm [44] and was calculated using an extinction coefficient of 26.40 M⁻^1^ cm⁻^1^. All activity is expressed as µM g⁻^1^ f.wt. min⁻^1^. The activity of peroxidase was determined using the assay described by [45]. The reaction was monitored by measuring the change in optical density at 470 nm for 1 min, and the activity was expressed as µM min⁻^1^ g⁻^1^ fresh weight.

### Extraction of total RNA, cDNA synthesis and RT-PCR:

Total RNA was extracted from 100 mg of the fully expanded leaves, then leaf samples were immediately snap-frozen in liquid nitrogen, stored at −80 °C, and then pulverized into a fine powder using a pre-chilled mortar and pestle under liquid nitrogen to prevent RNA degradation. Extraction was performed utilizing the Qiagen RNase Mini Kit. Total RNA was extracted using the Qiagen RNeasy Mini Kit (Qiagen, Germany) following the manufacturer's protocol. cDNA was synthesized in a 20 μl reaction volume using M-MLV reverse transcriptase (Promega, USA), 5 × RT buffer, 10 mM dNTPs, 50 ng/μl oligo(dT)18 primer, 40 U/μl RiboLock RNase inhibitor, and 200 U/μl M-MLV RT enzyme. The thermal cycling conditions were: 5 min at 65 °C (primer annealing/DNA denaturation), 60 min at 42 °C (reverse transcription), and 10 min at 95 °C (enzyme inactivation). Quantitative real-time PCR was conducted in triplicate using Maxima SYBR Green/ROX qPCR Master Mix (Thermo Fisher Scientific, USA) in a 25 μl reaction volume containing 1 μl cDNA (1:10 dilution), 12.5 μl 2 × Master Mix, 0.5 μM each primer (*CAT2, SOD1, CHLH* and *DREB1*; Table 1), and nuclease-free water. Thermal cycling on the Rotor-Gene Q (Qiagen, Germany) consisted of 95 °C for 10 min, followed by 40 cycles of 95 °C for 15 s and 60 °C for 60 s (combined annealing/extension). Relative gene expression was quantified using the 2^(-ΔΔCt) method with ACTIN as the reference gene [46].

**Table 1:** Specific sequences of primers used in this study

Gene name	Abbreviation	Forward (F) and reverse (R) primer 5′–3′
*Reference gene*	*ACTIN*	F: 5′-GGTAACATTGTGCTCAGTGGTGG-3′ R: 5′-AACGACCTTAATCTTCATGCTGC-3′
*Superoxide dismutase1*	*SOD1*	F: 5′-TTCGAAGGGCAGAGTCGAAG-3′ R: 5′-CAAACGAGGCATGCCCAAAA-3′
*Catalase2*	*CAT2*	F:5′-TGACTGCGGATTCGAGCAA-3′ R: 5′-AACGCAAGAAGAGGGGTTGA-3′
*Magnesium chelatase*	*CHLH*	F:5′-GGTGAAAATCGTGGAGGAAA-3′ R: 5′-ATGTCGCCTCTCAATCCATC-3′
*Dehydration-Responsive Element Binding 1*	*DREB1*	F:5′-ATGAGCACAAAATCAGTTGTAG-3′ R: 5′-TCAGATGGAGAAGCTCCA-3′

### Statistical analysis

The findings are reported as the means of three replicates, and the standard deviation (SD) was computed. One-way ANOVA was employed for statistical analysis to identify significant differences among treatments. Analyses were conducted using XLSTAT software (version 2014.5.03), with a significance threshold set at Tukey's test, p < 0.05.

## Results

### Characterization of CAp NPs

#### UV–visible spectrum of CAp NPs

**Fig. 2:**
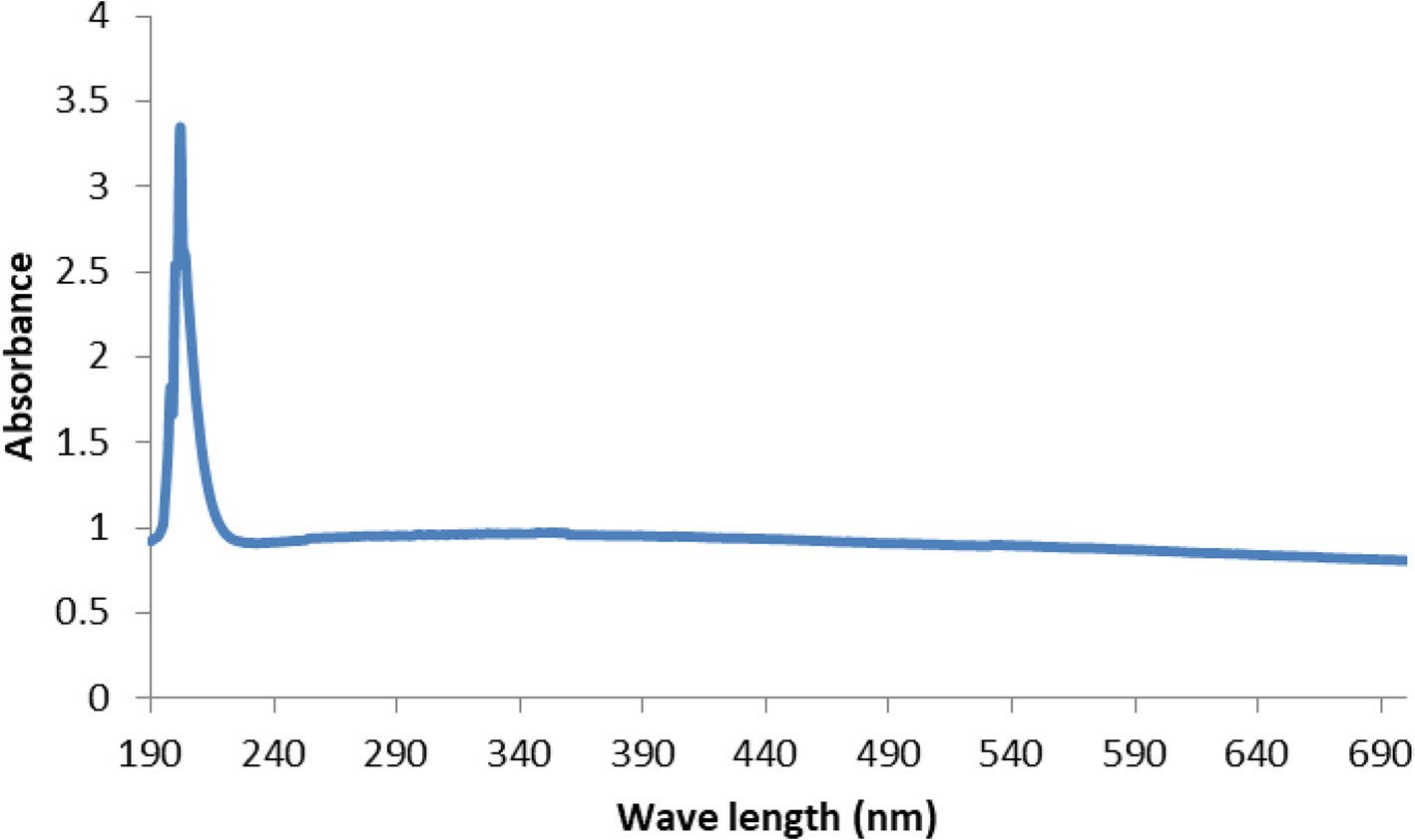
UV–Vis spectrum of biosynthesized CAp NPs

The UV–Vis spectrum of biosynthesized CAp NPs exhibited distinct single-band absorption with a maximum peak at 202 nm in the range of 200–800 nm, as shown in Fig. 2, confirming the biogenic fabrication of CAp NPs.

#### Transmission electron micrograph of CAp NPs

**Fig. 3:**
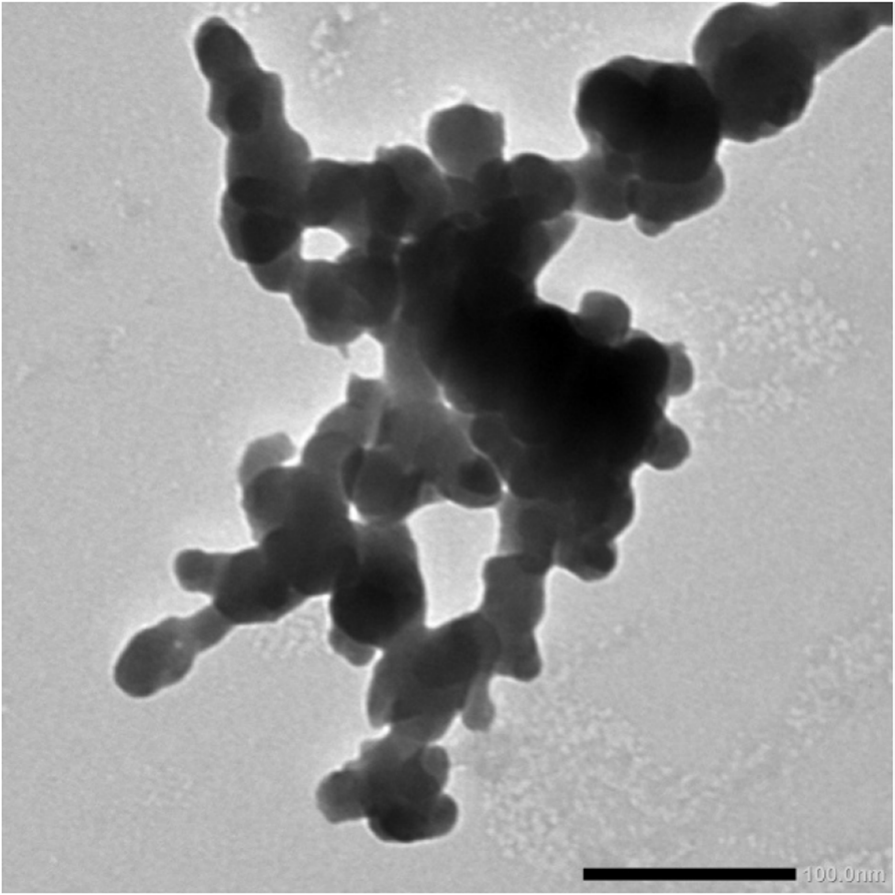
TEM micrographs of biosynthesized CAp NPs

For further characterization of CAp NPs, their size and morphology were examined using transmission electron microscopy (TEM). The TEM micrographs (Fig. 3) confirmed the formation of spherical CAp NPs with an average diameter of 18.5 ± 3.7 nm.

#### Fourier transforms infrared spectroscopy of CAp NPs

**Fig. 4:**
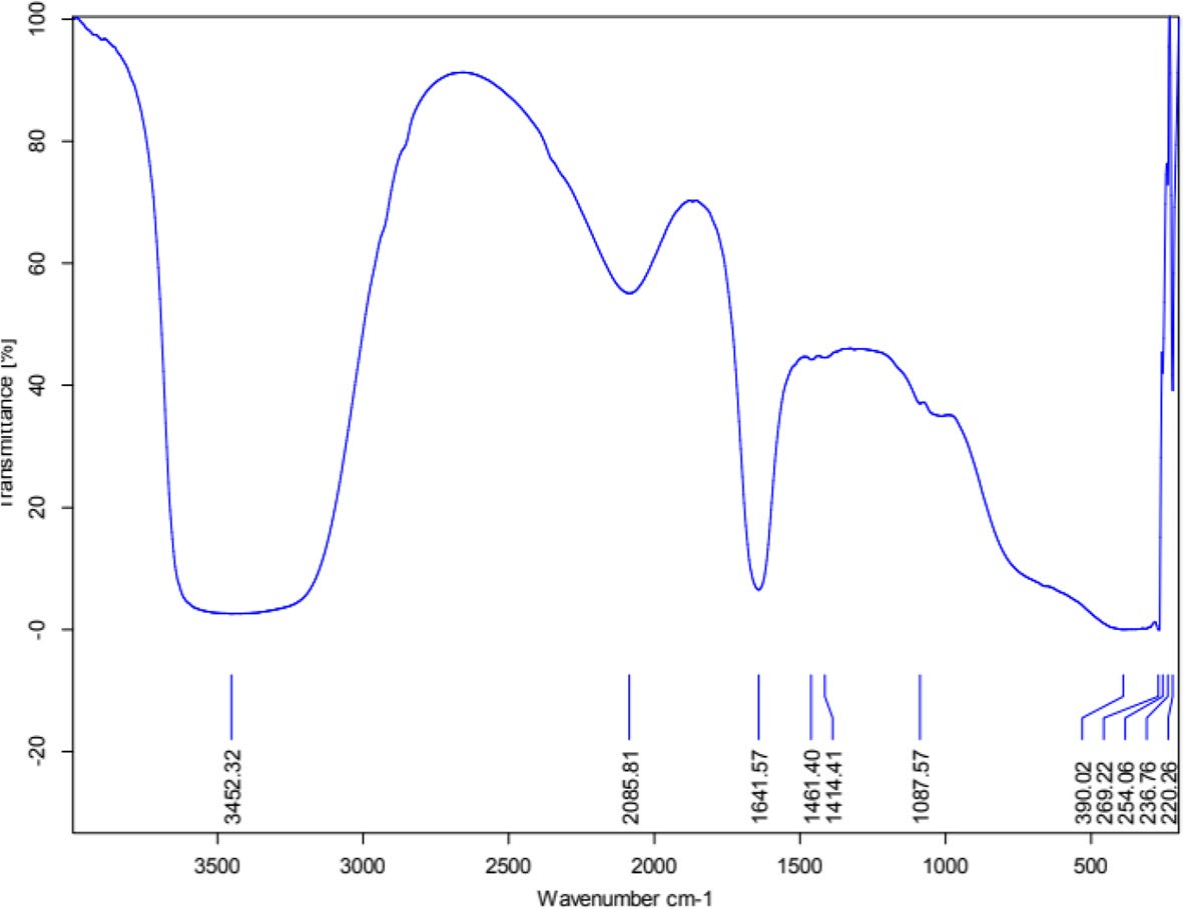
FT-IR analysis of biosynthesized CAp NPs

To elucidate the functional groups involved in the capping and stabilization of the CAp NPs, FT-IR analysis was conducted (Fig. 4). The resulting spectra (400 to 4000 cm-1) displayed characteristic absorption bands. The CAp NP spectrum showed distinct absorption peaks at wavenumbers 3452, 2085, 1641, 1461, 1414, 1087, 390, 269, 254, 236 and 220 cm-1.

#### 4-X-ray diffraction of CAp NPs

**Fig. 5:**
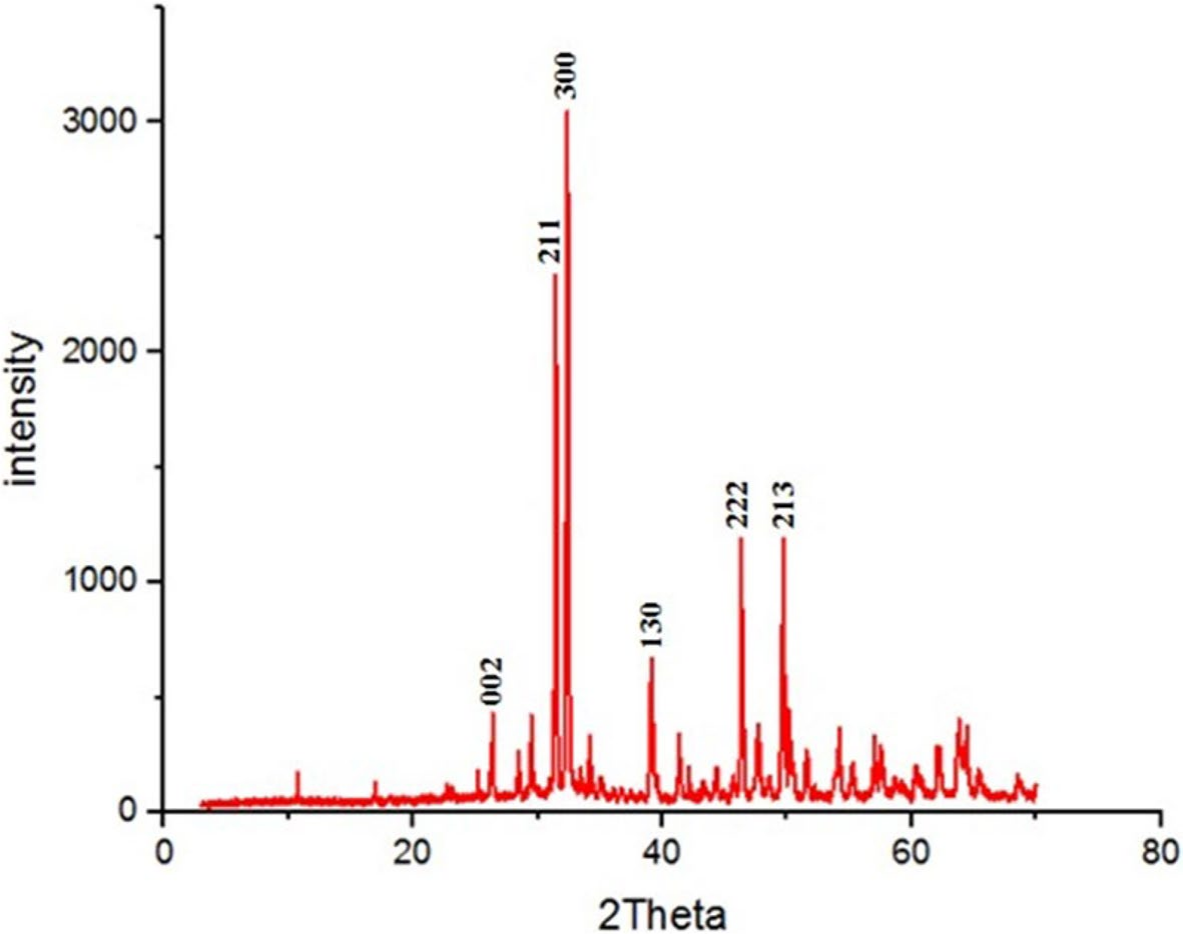
XRD spectra of biosynthesized CAp NPs

The XRD pattern revealed sharp and intense peaks with 2θ values of 26.3°, 32.2°, 33.364°, 39.06°, 46.25° and 49.5° corresponding to the (002), (211), (300), (130), (222), and (213) planes, respectively. These peaks are consistent with the hexagonal crystalline phase of chlorapatite (CAp NPs) as defined by JCPDS (Reference: 01–083–9127) as shown in Fig. 5.

#### Effect of CAp NPs on some growth parameters

**Fig. 6:**
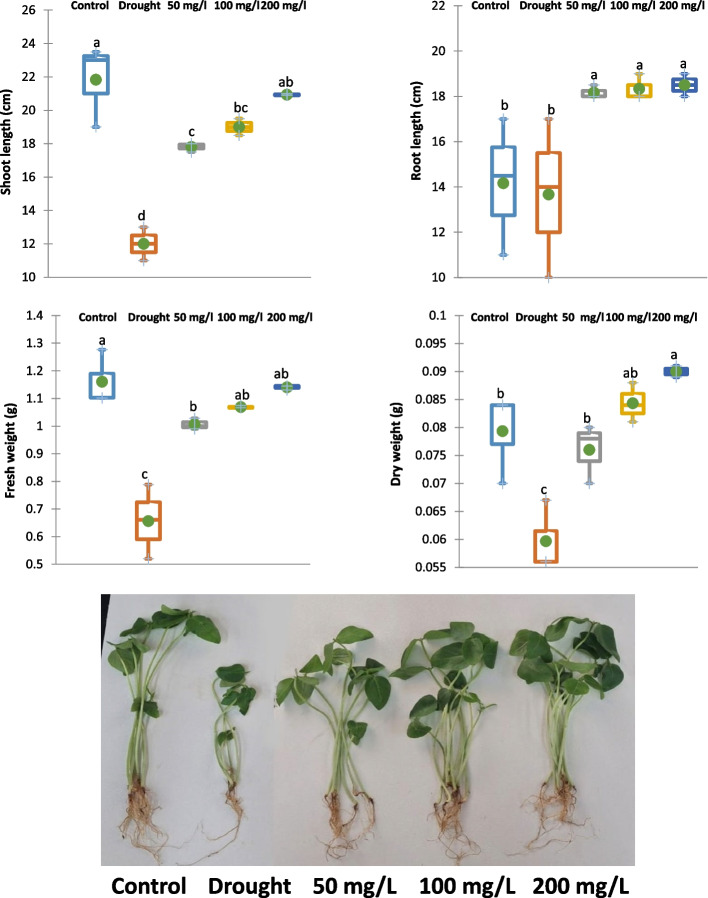
Effects of irrigation with CAp NPs (50, 100 or 200 mg/L) on the growth parameters of 15-d old cowpea plants

The application of 30% water field capacity (WFC) resulted in a significant reduction in shoot length (45%), root length (4%), fresh weight (44%) and dry weight (22%) of 15-d old cowpea plants relative to the control cowpea plant samples as shown in Fig. 6. Furthermore, irrigation with 50, 100 or 200 mg/L CAp NPs alleviated the drought effects, promoting increases in shoot length, root length, fresh weight and dry weight of 15-d old cowpea plants by 48, 58 and 74%, respectively for shoot length, 34, 35 and 36%, respectively for root length, 55, 63 and 75%, respectively for fresh weight and 32, 43 and 48%, respectively for dry weight compared to drought-stressed cowpea plants.

#### Soil chemical analysis

Soil pH values significantly increased in response to the high CAp NPs treatments. At the end of the experiment, the pH ranged from 6.67 ± 0.20 to 7.51 ± 0.08 for 50 mg/l, and 200 mg/l CAp NPs (Table 2). Moisture content and soil electrical conductivity (EC) significantly increased toward high CAp NP treatments. Moreover, after CAp NP treatment, OM contents significantly increased from 2.65 ± 0.06 to 2.99 ± 0.02% in CAp NPs 50 mg/l, and CAp NPs 200 mg/l; respectively. Compared with that in the control treatment, the soil available phosphorus in all CAp NP treatments significantly increased (Table 2). Total N content significantly increased in CAp NPs at 200 mg/l (0.17 ± 0.02%) compared with that in the control (0.09 ± 0.01%).

**Table 2:** Chemical analysis of the soil exposed to different treatments

**Parameters**	**IC**	day 15				
		**Control**	**Drought**	CAp NPs** 50 mg/l**	CAp NPs** 100 mg/l**	CAp NPs** 200 mg/l**
pH	6.42 ± 0.03^c^	6.54 ± 0.28^bc^	6.32 ± 0.09^c^	6.67 ± 0.20^bc^	7.02 ± 0.31^ab^	7.51 ± 0.08^a^
Moisture%	72.28 ± 2.12^ab^	73.6 ± 2.06^a^	54.24 ± 3.59^c^	62.56 ± 1.63^b^	65.55 ± 1.61^b^	68.32 ± 0.95^ab^
EC (mS/cm)	0.81 ± 0.03^b^	0.82 ± 0.03^ab^	0.77 ± 0.04^b^	0.82 ± 0.06^ab^	0.87 ± 0.02^ab^	0.92 ± 0.01^a^
OM%	3.22 ± 0.2^a^	3.31 ± 0.18^a^	2.34 ± 0.07^c^	2.65 ± 0.06^bc^	2.73 ± 0.18^b^	2.99 ± 0.02^ab^
P (ppm)	16.71 ± 0.35^de^	16.96 ± 0.07^d^	15.83 ± 0.50^e^	19.96 ± 0.33^c^	21.28 ± 0.39^b^	23.76 ± 0.48^a^
N %	0.09 ± 0.01^c^	0.11 ± 0.02^bc^	0.1 ± 0.015^c^	0.13 ± 0.01^ab^	0.15 ± 0.01^ab^	0.17 ± 0.02^a^

IC: Initial control (Day 0), Means that do not share a letter are significantly different (Tukey's test, p < 0.05)

#### Total abundance and species composition of oribatid mites

**Fig. 7:**
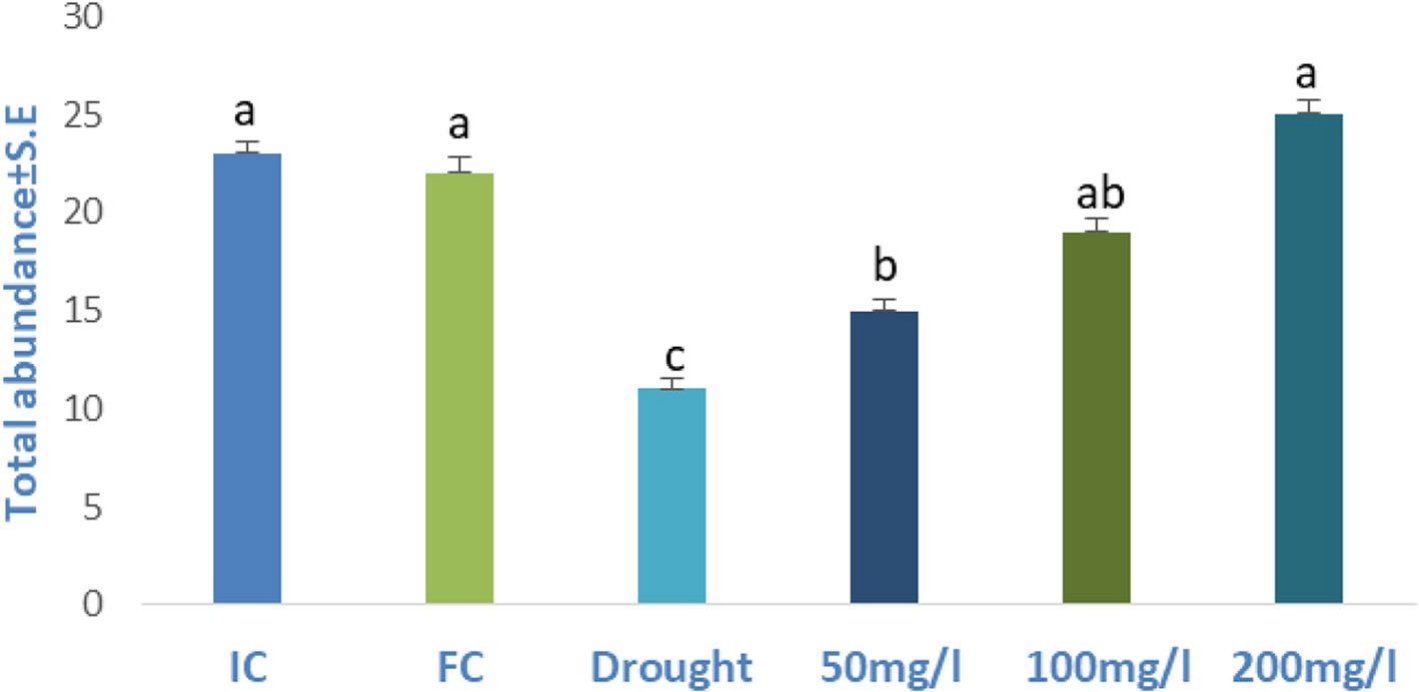
Total abundance of soil oribatid mites at the beginning and end of the experiment across different treatments, IC: initial control on day 0 (at the beginning of the experiment), FC: final control (at the end of the experiment) on day 15

Seven species of oribatid mites belonging to seven genera and six families were extracted from the investigated treatments, as shown Table 3. Total abundance at the start of the experiment was 23 ± 0.64. At the end of the experiment, total abundance in the control plots was 22 ± 0.79 individuals, (Fig. 7). Total abundances of oribatid mites in the different treatment groups were generally lower than in those the controls except in CAp NP 200 mg/l treatment. Additionally, CAp NP application significantly affected total oribatid abundance.

**Table 3:** List of species of oribatid mites, their relative contributions, and dominance classification under different treatments

***Species***	**IC**	day 15				
		**Control**	**Drought**	**CAp NPs 50 mg/l**	**CAp NPs 100 mg/l**	**CAp NPs 200 mg/l**
*Lohmannia hispaniola*	13.04 B	13.63 B	-	20 B	15.78 B	12.5 B
*Tectocepheus sarekensis*	8.69 C	13.63 B	-	13.33 B	10.52 B	4.16 D
*Scheloribates laevigatus*	26.08 B	27.27 B	27.27 B	26.66 B	26.31 B	25 B
*Zygoribatula undulata*	21.73 B	27.27 B	18.18 B	20 B	26.31 B	25 B
*Xylobates capucinus*	13.04 B	4.54 D	27.27 B	20 B	10.52 B	12.5 B
*Lamellobates hauseri aegypticus*	13.04 B	9.09 C	27.27 B	-	5.26 C	12.5 B
*Anachipteria aegyptiaca*	4.34 D	4.54 D	-	-	5.26 C	8.33 C

IC: Initial control (Day 0), %; relative dominance in community; dominance class is indicated by capital letters, B dominant; 10–30% of individuals, C sub-dominant;5–10%of individuals, and D minor;1–5% of individuals

#### Species diversity of oribatid mites

Two diversity indices (Simpson's D and Shannon's H′) were used to detect the impact of CAp NP application. The number of species decreased from 7 at the beginning of the experiment to 4 under drought. Simpson's D and Shannon's H′ values in the majority of treatments tended to decrease in comparison to the controls. At the start of the experiment, Shannon's diversity index (H') reached 1.80 and evenness (E) was 0.92, whereas Simpson's index (D) reached 5.68 and equitability (J) was 0.81. At the end of the experiment, the highest H' values were recorded in CAp NP 200 mg/l treatment (1.805), whereas the lowest values were recorded in drought treatment (1.371). Similarly, the highest D values were detected in IC treatment group (5.68). On the other hand, the lowest D values were recorded under drought (3.90). Equitability (J) and evenness (E) values differed across different treatments Table 4.

**Table 4:** Species diversity and equitability values of soil oribatid mites in different treatments

**Diversity index**	**IC**	day 15				
		**Control**	**Drought**	**CAp NPs 50 mg/l**	**CAp NPs 100 mg/l**	**CAp NPs 200 mg/l**
**Species richness**	7	7	4	5	7	7
**D**	5.68	5.04	3.90	4.78	5.23	5.54
**J**	0.81	0.72	0.97	0.95	0.74	0.79
**H**	1.80	1.744	1.371	1.583	1.770	1.805
**E**	0.92	0.89	0.98	0.98	0.90	0.92

*IC* Initial control (Day 0). D: Simpson’s index, J: Equitability, H: Shannon- wiener index, and E: Evenness

#### Effect of CAp NPs on oxidative stress

**Fig. 8:**
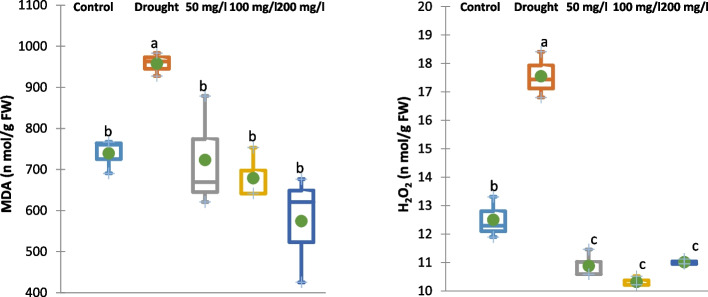
Effects of irrigation with CAp NPs (50, 100 or 200 mg/L) on oxidative stress markers (MDA and H_2_O_2_) in 15-d old cowpea plants

Application of 30% of WFC led to a significant accumulation of reactive oxygen species, increasing the contents of malondialdehyde (MDA) and hydrogen peroxide (H₂O₂) in 15-d old cowpea plants by 30 and 40%, respectively compared to the control cowpea plant samples as showed in Fig. 8. Furthermore, irrigation with 50, 100 or 200 mg/L CAp NPs substantially alleviated this oxidative damage, notably diminished the contents of MDA and H₂O₂ in 15-d old cowpea plants by 25, 29 and 40%, respectively for MDA content and 38, 41 and 37%, respectively, for H₂O₂ in comparison with those in drought-stressed cowpea plants.

#### Effect of CAp NPs on Osmoprotectants

**Fig. 9:**
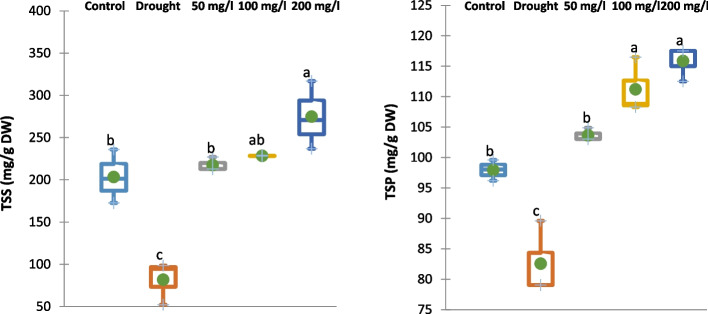
Effects of irrigation with CAp NPs (50, 100 or 200 mg/L) on the contents of TSS and TSP in 15-d old cowpea plants

Application of 30% of WFC significantly decreased the total soluble sugar (TSS) and total soluble protein (TSP) contents of 15-d old cowpea plants by 60 and 16%, respectively compared to the control cowpea plant samples as shown in Fig. 9. On the contrary, irrigation with 50, 100 or 200 mg/L CAp NPs notably enhanced the contents of TSS and TSP of 15-d old cowpea plant by 167, 179 and 236%, respectively for TSS and 26, 35 and 40%, respectively for TSP compared to drought-stressed cowpea plants.

### Effect of CAp NPs on non-enzymatic and enzymatic antioxidants

#### Non-enzymatic antioxidants

**Fig. 10:**
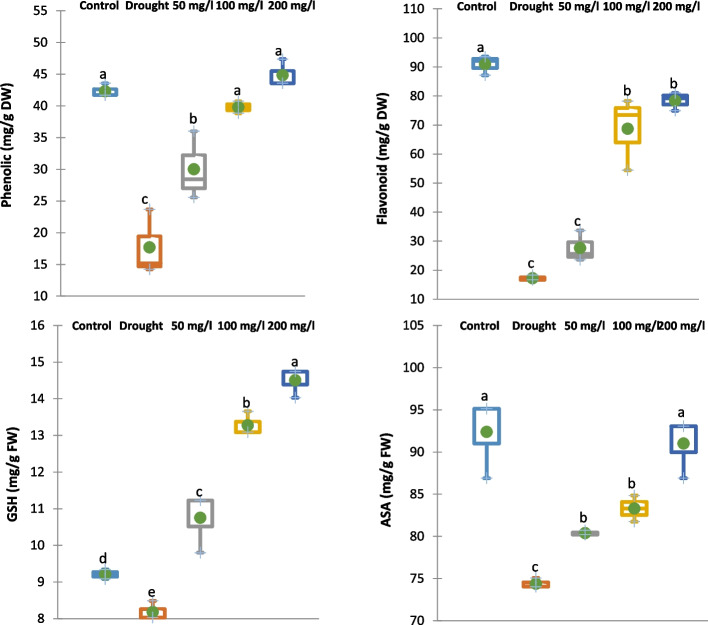
Effects of irrigation with CAp NPs (50, 100 or 200 mg/L) on the content of total phenolic, flavonoids, ASA, and GSH of 15-d old cowpea plants

Application of 30% of WFC significantly diminished the content of total phenolic, flavonoids, ascorbic acid (ASA), and reduced glutathione (GSH) of 15-d old cowpea plants by 58, 81, 20 and 11%, respectively, relative to well-watered control samples as represented in Fig. 10. Meanwhile, irrigation with 50, 100 or 200 mg/L CAp NPs markedly enhanced the levels of these antioxidants in the stressed plants by 70, 126 and 155%, respectively for total phenolic, 60, 299 and 356%, respectively for flavonoids, 8, 12 and 22%, respectively for ASA and 30, 61 and 77%, respectively for GSH compared to drought-stressed cowpea plants.

#### Enzymatic antioxidants

**Fig. 11:**
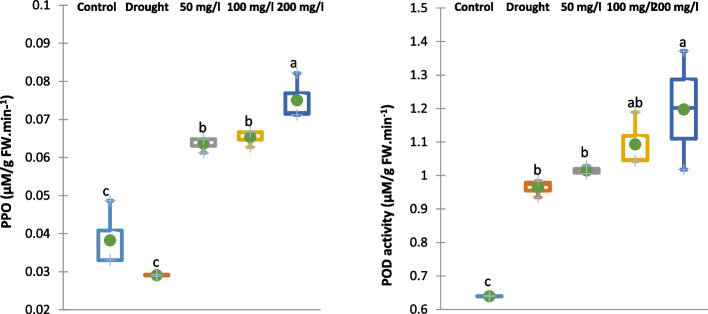
Effect of irrigation with CAp NPs (50, 100 or 200 mg/L) on the activity of PPO and POD in15-d old cowpea plants

Application of 30% of WFC significantly diminished the activity of polyphenol oxidase (PPO) of 15-d old cowpea plant by 24% while increased the activity of peroxidase (POD) by 52% compared to the control cowpea plant samples as represented in Fig. 11. Meanwhile, irrigation with 50, 100 or 200 mg/L CAp NPs notably improved the PPO and POD activities of 15-d old cowpea plant by 107, 108 and 159%, respectively for PPO and 5, 14 and 24%, respectively, for POD compared to drought-stressed cowpea plants.

#### Effect of CAp NPs on relative gene expression

**Fig. 12:**
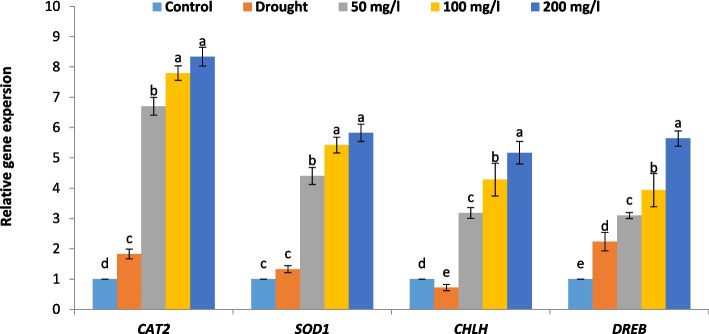
Effect of irrigation with CAp NPs (50, 100 or 200 mg/L) on relative gene expression of 15-d old cowpea plants

Application of 30% of WFC significantly increased the relative gene expression of *CAT2*, *SOD1* and *DREB1* of 15-d old cowpea plant while diminished the relative gene expression of *CHLH* compared to the control cowpea plant samples as represented in Fig. 12. Meanwhile, irrigation with 50, 100 or 200 mg/L CAp NPs notably improved the relative gene expression of *CAT2*, *SOD1*, *CHLH* and *DREB1*, particularly with 200 mg/L CAp NPs, compared to drought-stressed cowpea plants.

## Discussion

Drought stress induces multifaceted damage to plants, undermining agricultural productivity worldwide by interfering with essential processes such as photosynthesis and nutrient assimilation. To combat this, nano-fertilizers represent a progressive solution that outperforms traditional chemical options by delivering nutrients more effectively, thereby improving crop resilience and output while lessening pollutant load [47]. Advancing this paradigm, green-synthesized nanoparticles stand out as powerful, low-cost nano-fertilizers with the potential to supersede conventional products, offering a direct pathway to mitigate the widespread problem of excessive agrochemical application [7, 13]. Therefore, greenery synthesized CAp NPs by using pectin extracted from *Ficus elastica* leaves, which was then used to evaluate the effectiveness of CAp NPs to ameliorate the cowpea plant's resistance to drought stress. The greenery synthesized CAp NP powder appeared pale yellow-green color and was characterized by using UV–Visible spectroscopy. The resulting UV–visible spectrum indicated that the CAp NPs had a peak at 202 nm in UV region. These results are in agreement with those obtained by [48, 49], who reported that apatite nanoparticles have absorption spectra between 200 and 220 nm. Additionally, TEM confirmed the formation of spherical CAp NPs with possessed a narrow size distribution, averaging 18.5 ± 3.7 nm. For further analysis, the XRD pattern was consistent with the hexagonal crystalline phase of CAp NPs, which is consistent with the results reported by [50, 51].

The stretching O–H peak appears in pectin extracted from *Ficus elastica* leaves spectrum as a very distinctive band extends from peak recorded at 3452 cm⁻^1^, characteristic of the polysaccharide backbone of pectin. The peak at 1641 cm⁻^1^ is attributed to the C = O stretching of the non-methylated carboxylic acid groups in pectin, which likely serve as the primary binding sites for Ca^2+^ ions. The 1414 cm^−1^ bands were assignable to CO_3_^2^ions in OH^−^ sites of the CAP crystals [52]. The presence of the peak at 1461 cm^−1^ is related to CO_3_^2^group and suggests that carbon from the organics doesnot pyrolyze completely and may instead dissolve into the CAp crystal [53]. The significant peak at 1087 cm⁻^1^ unambiguously confirms the stretching of apatite, hence validating the production of nanoparticles [54, 55]. These findings validate the effectiveness of pectin as a capping and templating agent, successfully coating and stabilizing the nanoparticles.

Severe water deficit (30% WFC) profoundly reduces cowpea biomass, significantly reducing shoot length, fresh weight, dry weight, and *CHLH* gene while root length is minimally affected, suggesting a drought-avoidance strategy where resource allocation favors root survival. Additionally, under drought, plants close their stomata to reduce water loss, which concurrently limits CO₂ uptake and photosynthesis. This reduces the production of sugars, directly impacting biomass accumulation and elongation growth [56, 57]. Meanwhile, irrigation with CAp NPs notably alleviated drought-induced growth inhibition. The dose-dependent increases in growth parameters and *CHLH* gene indicate that CAp NPs can significantly enhance plant resilience to drought stress. The beneficial effects of CAp NPs are likely due to improved nutrient availability (especially phosphorus and calcium). Phosphorus is crucial for energy transfer (ATP), photosynthesis, and membrane integrity. Drought stress often impairs phosphate uptake from dry soil. CAp NPs may provide a direct, efficient P source, supporting energy metabolism and recovery [58]. The delivered calcium (Ca^2^⁺) from CAp is a key signaling ion for stomatal closure. Optimal Ca^2^⁺ signaling might help plants regulate stomata more efficiently under drought [59, 60]. Similar to CAp NPs, the activated mechanisms may involve the upregulation of the *CHLH* gene. This gene is critical for chlorophyll biosynthesis, as it catalyzes the insertion of Mg^2^⁺ into protoporphyrin the first dedicated step in the pathway [61]. To enhance adaptation to fluctuating land environments, particularly drought and salinity, the CHLH protein has evolved multifunctional roles in chlorophyll biosynthesis, retrograde signaling, and abscisic acid responses, while also integrating into broader chloroplast-derived and hormonal signaling pathways [61, 62].

The observed amelioration of drought stress in cowpea plants by CAp NPs is likely underpinned by significant improvements in key soil physicochemical properties, as revealed in this study. The dose-dependent increase in soil pH towards neutrality is consistent with the known alkaline nature of apatite, which can buffer soil acidity and enhance the availability of essential nutrients such as phosphorus [9]. This is directly corroborated by the significant rise in available phosphorus in treated soils, providing a direct nutritional pathway for plant growth promotion under stress [63]. Furthermore, the substantial increase in soil moisture content, particularly at the highest NP concentration (200 mg/L), offers a direct mechanism for the observed mitigation of drought effects; improved water retention reduces plant water deficit, explaining the recovery of growth parameters and reduction in oxidative stress markers. The concurrent increases in electrical conductivity (EC), organic matter, and total nitrogen suggest that apatite NP application stimulated microbial activity and nutrient cycling, creating a more fertile rhizosphere [24]. Importantly, the moderated impact on oribatid mite diversity at 200 mg/L may be linked to overall soil conditioning, as a more stable and resource-rich environment can support microarthropod communities. These findings align with and extend previous research on nano-enhancers of soil quality [24], confirming that the benefits of CAp NPs are not solely direct plant interactions but are critically mediated through the improvement of the soil habitat, which synergistically enhances plant drought tolerance and supports soil ecosystem stability.

The imposition of drought stress induces stomatal closure, thereby restricting the availability of CO_2_ within the leaf and suppressing carbon fixation. Consequently, chloroplasts are subjected to excess excitation energy, leading to an elevated production of ROS, namely, H₂O₂, superoxide, and hydroxyl radicals [64]. The excessive production of ROS, in response to various environmental stresses, leads to membrane disfunction and causes cell death [65]. The present study's findings demonstrated the elevated MDA and H₂O₂ contents of 15-d old cowpea plant in response to application of 30% WFC. This refers to major lipid peroxidation and redox imbalance resulting from the accumulation of ROS which damages cellular integrity [66]. Furthermore, irrigation with chlorapatite nanoparticles (CAp NPs) reduced the contents of MDA and H₂O₂ compared to drought-stressed plants. This reduction suggests that CAp NPs mitigate oxidative stress by enhancing the plant’s antioxidant capacity, likely through improved nutrient status (especially phosphorus and calcium) and stimulation of antioxidant enzyme activity [67]. The dose-dependent decrease in MDA and H₂O₂ contents indicates that higher concentrations of CAp NPs are more effective at alleviating oxidative damage, supporting their role in protecting cowpea plants from drought-induced oxidative stress.

Additionally, the result provides crucial biochemical evidence that CAp NPs not only mitigate oxidative damage but also actively enhance the plant's osmotic adjustment and metabolic resilience under drought. The severe reduction in total soluble sugars and total soluble proteins under 30% WFC reflects a metabolic crisis where drought impairs photosynthesis and promotes protein degradation, depleting the essential osmoprotectants and nitrogen reserves needed to maintain cell turgor and enzymatic function [56, 57]. Conversely, the profound, dose-dependent enhancement of these compounds with CAp NP application, particularly the extraordinary increase in TSS demonstrates a powerful restorative mechanism. This likely occurs through multiple pathways: the improved phosphorus nutrition from CAp supports ATP synthesis, energizing photosynthesis and sugar production; the amelioration of oxidative stress (as shown by reduced H₂O₂ and MDA) protects photosynthetic machinery and cellular enzymes, preserving protein integrity; and the calcium ions may act as signaling molecules to upregulate biosynthetic pathways for osmolyte production [68–70]. The resulting accumulation of TSS and TSP directly contributes to lowering cellular osmotic potential, improving water retention, stabilizing membranes and enzymes, and providing metabolic substrates for recovery, thereby biochemically underpinning the observed restoration of growth, biomass, and overall drought tolerance.

The current study's results show that application of 30% WFC diminished both enzymatic and non-enzymatic antioxidants (including flavonoids, phenolic, ASA, GSH, POD, and PPO) and relative gene expression of *CAT2* and *SOD1* in cowpea plants. This reflects the typical response to drought stress, where the synthesis and accumulation of antioxidant metabolites are suppressed, making plants more susceptible to oxidative damage [71]. In contrast, irrigation with CAp NPs markably restored and even enhanced these antioxidant levels in drought-stressed cowpea plants. This is a classic stress response. POD is a frontline enzyme that uses various compounds (like phenolics) to detoxify H₂O₂, making its increased activity a direct adaptive mechanism to drought-induced oxidative stress [72]. Additionally, PPO is involved in phenolic metabolism and can have pro-oxidant effects. Its suppression might be a plant response to conserve phenolic substrates or avoid further oxidative damage [73]. Also, the enhanced relative gene expression of *SOD1* and *CAT2* could support the hypothesis that H_2_O_2_ results from oxygen free radicals including O2• − [74, 75]. According to reports, plants become more resilient to oxidative stress when the majority of *SOD1* and *CAT2* genes are over expressed [14]. Such improvements are consistent with studies showing that CAp NPs can mitigate drought stress by improving nutrient uptake, enhancing the biosynthesis of secondary metabolites, and boosting antioxidant defenses [22, 67].

The *DREB* gene family plays a critical role in cowpea response to drought stress. In cowpea, the DREB transcription factor has been identified and characterized as a key regulator of drought tolerance [76]. Consistent with these findings, our study found that drought stress (30% WFC) significantly increased the relative gene expression of *DREB1* in 15-day-old cowpea plants. This induction is part of the adaptive mechanism of cowpea to cope with drought [76]. Notably, irrigation with CAp NPs improved the relative expression of *DREB1*, especially at the highest concentration (200 mg/l), compared to drought-stressed plants. This suggests that CAp NPs not only alleviate drought stress but also amplify the plant's molecular defense by boosting the expression of key stress-responsive genes like *DREB1*. This amplification of the plant's molecular defense by CAp NPs likely explains the observed physiological resilience, as DREB proteins are known to orchestrate downstream processes like osmotic adjustment through osmoprotectant accumulation (e.g., proline and sugars) and the bolstering of antioxidant systems [77]. Kumar et al. [78] reported that transgenic cowpea plants overexpressing *DREB* genes exhibit enhanced osmotic adjustment, antioxidant defense, and improved photosynthetic activity under drought conditions, demonstrating the importance of DREB transcription factors in drought resilience.

## Conclusion

In conclusion, this study demonstrates that biogenically synthesized chlorapatite nanoparticles (CAp NPs) serve as an effective nanopriming agent for enhancing drought tolerance in cowpea. The optimal concentration of 200 mg/L CAp NPs significantly mitigated the adverse effects of water deficit by improving plant growth, enhancing osmotic adjustment and antioxidant capacity, reducing oxidative damage, and modulating the expression of key stress-responsive genes, specifically *CAT2, SOD1, DREB*, and *CHLH*. Concurrently, this treatment showed a comparatively less disruptive effect on soil health, as indicated by the higher diversity and evenness of oribatid mite communities relative to other drought-exposed groups. These findings highlight the dual benefit of CAp NPs in promoting plant resilience under abiotic stress while partially preserving soil microarthropod biodiversity, positioning them as a promising sustainable tool for agricultural applications.

## Data Availability

All data supporting the findings of this study are already presented in this published manuscript.

## Abbreviations

CAp NPs: Chlorapatite nanoparticles
SOD1: Superoxide dismutase1
CAT2: Catalase2
CHLH: Magnesium chelatase
DREB1: Dehydration-Responsive Element Binding 1
XRD: X-ray diffractometer
TEM: Transmission electron microscopy
FTIR: Fourier Transform Infrared Spectroscopy
POD: Peroxidase
PPO: polyphenol oxidase
ASA: Ascorbic acid
TSS: Total soluble sugars
TSP: Total soluble proteins
H_2_O_2_: Hydrogen peroxide
MDA: Malondialdehyde
